# A rapid narrative review of the clinical evolution of psychedelic treatment in clinical trials

**DOI:** 10.1038/s44184-024-00068-9

**Published:** 2024-07-02

**Authors:** Ronit Kishon, Nadav Liam Modlin, Yael M. Cycowicz, Hania Mourtada, Tayler Wilson, Victoria Williamson, Anthony Cleare, James Rucker

**Affiliations:** 1 Department of Psychiatry, Columbia University, New York, NY USA; 2 New York State Psychiatric Institute, New York, NY USA; 3 The Institute of Psychiatry, Psychology and Neuroscience, King’s College London, London, UK; 4 South London and Maudsley NHS Foundation Trust, Maudsley Hospital, London, UK; 5 Department of Clinical Psychology, Teachers College, Columbia University, New York, NY USA; 6 Department of Experimental Psychology, Anna Watts Building, University of Oxford, Oxford, UK

**Keywords:** Psychology, Medical research

## Abstract

Pre-prohibition psychedelic research with complex psychiatric patients generated a wealth of treatment methodologies and practices, providing invaluable clinical insights pertaining to the medical administration of psychedelics in various mental health diagnoses. Building upon these early studies, which lack the rigor and research tools available today, contemporary psychedelic research has focused on investigating the safety and efficacy of psychedelics in randomized controlled trials via psychometric measures and symptom assessments. Both then and now, the treatment context and the role of clinicians in psychedelic treatment has been recognized as an essential feature for positive patient outcomes. To broaden the knowledge base of modern psychedelic research and support the training of clinicians conducting medically supervised psychedelic research studies, this paper provides a review of pre-prohibition clinical research narratives pertaining to the phenomenology of psychedelic treatment and the role of the non-pharmacological treatment factors in the patient experience. Lastly, this paper explores a range of clinician perspectives and psychological interventions employed in pre-prohibition psychedelic research to inform future research directions and best practice guidelines.

## Introduction

Medical reports concerning the effects of psychedelics such as 3,4,5-trimethoxyphenethylamine (mescaline) date back to the 1890s, noting their profound effects on perception, cognition, and potential ‘value for psychology’^1^. However, scientific research remained sporadic until the synthesis of lysergic acid diethylamide (LSD) by Dr. Hofmann in 1938^2,3^ which marked the beginning of the pre-prohibition era of psychedelic research. During this prolific period of clinical experimentation and scientific inquiry, clinicians and researchers studied the use of LSD and other classical psychedelics in the treatment of a range psychiatric disorders^4^. However, increased recreational use, concerns about safety and abuse, and shifting political attitudes led to the prohibition of psychedelics by the late 1960s and early 1970s.

Clinically, the unique phenomenology of LSD inspired hypotheses regarding its potential psychiatric applications^5^. The pharmaceutical company Sandoz pursued research in this domain by distributing LSD to investigators worldwide free of charge. Sandoz recommended its use as an aid to psychotherapy, referred to as the “psycholytic”^6^. The treatment involved administrating small, gradually increasing doses of psychedelics alongside established psychoanalytic treatment, to patients diagnosed with difficult-to-treat conditions^7^. Subsequently, many pre-prohibition, predominantly psychoanalytically oriented psychiatrists, noted psychedelics’ therapeutic potential^8^, highlighting their capacity to stimulate the emergence of unconscious material by enhancing the transference and eliciting highly associative states^9^.

As the clinical use of psychedelics increased across residential^9,10^ and outpatient settings^11^, in individual^12^ or group-based^13^ treatment protocols (Table 1), psychedelics appeared to challenge the prevailing psychoanalytic theories regarding the etiology of mental illness. LSD’s psychosis-like effects led some to propose biological models where endogenous compounds similar to mescaline and LSD caused psychotic disorders^14^, challenging traditional psychoanalytic conceptualizations of mental illnesses^15^ and adding to the evolving field of neuropsychiatry. This paradigm shift enabled the introduction of the first antipsychotic in 1952, superseding psychoanalytic schizophrenia treatment^16^. Further, LSD’s structural serotonin resemblance sparked hypothesizing in 1954 that serotonin dysfunction played a pathogenic role in affective illnesses^17^, shifting perspectives further toward biological frameworks. However, despite these novel perspectives, pre-prohibition researchers consistently emphasized the significance of psychological interventions to positive treatment outcomes^18,19^.

**Table 1:** Summary literature of pre-prohibition era

Study	Patient demographics	Treated disorder(s)	Psychedelic used/dosage	Therapy type	Treatment duration	Control group	Outcome measures
Whitaker^31^	*N* = 100 (*f* = 49)	Wide of treatment-resistant conditions, including schizophrenia, recurrent depression, and personality disorders	LSD (100–250 μg)	LSD integrated with unspecified psychotherapy	Varied number of LSD and psychotherapy sessions.	Yes	Symptoms reported by patient, and clinical assessment by therapist
Kast^59^	*N* = 128	Terminal metastatic malignant disease	LSD (100 μg)	None	One LSD session	None	Pain intensity, affective changes, approach to illness and death, sleep patterns, visual distortions/hallucinations, fear/panic reactions
Chwelos et al.^21^	*N* = 24, *N* = 16	Alcoholism, with character disorders, psychopaths, borderline, or actual psychoses	LSD (200–400 μg) at times mescaline (200 μg or more)	LSD session followed by AA meetings	One LSD session, and a few integration sessions, all in one week	None	Reduction in alcohol consumption
Chandler & Hartman^11^	*N* = 110 (*f* = 48) Age: 15–62	Psychoneurosis, addiction, personality, and sociopathic disorders	LSD-25, baseline dosages 25 μg or 50 μg or larger in later sessions	LSD session is a facilitating agent in psychotherapy	7–8 LSD sessions (each 4–4.5 h)	None	Therapist clinical assessment based on patients’ subjective reports
Smith^32^	*N* = 24	Alcoholism, psychopathy, and character disorders	LSD (200–400 μg) or mescaline	Psychotherapy aided by occupational and recreational therapy, LSD sessions as an adjunct	One LSD or mescaline session 2–4 weeks after initial treatment, follow-up 2 months to 3 years.	None	Alcoholics Anonymous assessments of alcohol consumption
Lewis & Sloane^9^	*N* = 23 (*f* = 8) Age: 18–64	Primarily obsessional illness, but few additional disorders	LSD (25–500 μg)	LSD sessions once or twice a week with psychodynamics interpretation	Few weeks to 4 months of LSD sessions. In some cases regular psychotherapy for up to a year	None	Therapist clinical assessment of symptoms
Eisner & Cohen^46^	*N* = 22	Neurotic depressions, anxiety states, character disorders, borderline schizophrenia	LSD 100–150 μg) increased incrementally.	LSD sessions with psychotherapy interpretation	4–5 weekly LSD sessions with supportive psychotherapy and a follow-up period of 6 to 17 months	None	Therapist clinical assessment of symptoms
Sandison et al. ^25^	*N* = 36	Psychoneuroses and related conditions	LSD (25–400 μg)	Psychotherapy aided by LSD sessions	One year with varying durations for individual patient	None	Improvement in psychoneurotic symptoms, recovery of repressed memories, mental state
Sandison^24^	*N* = 36	Neuroses, obsessional neurotics, depressive states	LSD (25–400 μg)	LSD-assisted psychotherapy, with Jungian analytical psychology	Use of LSD over 8 months	None	Improvement in recovery of repressed memories, changes in mental states, increase in patient’s faith, and in their artistic inclination
Jensen^10^	*N* = 45	Alcoholism	LSD (200 μg)	Inpatient individual and group therapy along with AA program, and a few LSD sessions	8 weeks treatment with 1 LSD session at the end. Follow up 6–18 months	Yes	Alcohol consumption – abstinence
Smart et al.^55^	*N* = 30 (*f* = 2)	Alcoholism	LSD (800 μg), or ephedrine (60 mg)	Inpatient treatment and one LSD/ephedrine session	One LSD /ephedrine session a week before the end of the treatment program	Yes	Duration and frequency of abstinence
Alnæs^30^	*N* = 20 patients *N* = 20 control All Females	neurosis, anxiety, compulsive neuroses	LSD (25–500 μg) with either Psilocybin (20–30 mg) or CZ-74	Psychoanalytically-oriented therapy with either psycholytic or psychedelic - peak sessions.	Varied number of LSD sessions as needed	None	A questionnaire evaluating changes in consciousness
Busch & Johnson^45^	*N* = 8 (4 Inpatient)	Mainly schizophrenia, psychoneurosis, and other disorders	LSD (30–40 μg)	Patients who had ongoing psychotherapy were chosen for one LSD session.	One LSD session (each 4–8 h)	None	Clinical assessment of an individual’s capacity to manage emotions, access memories, and cultivate insights concerning childhood trauma
Pahnke^84^	*N* > 300	Alcoholism, neuroses, narcotic, addiction, psychological distress (cancer patients)	LSD (200–450 µg) Dosages varied based on psychotherapy.	Psycholytic therapy, psychedelic-chemotherapy, and psychedelic-peak therapy	Varied, some studies had multiple sessions over a long term	Yes	Subjective improvement, Psychological and social measures, psychedelics safety measure in cancer patients
MacLean et al.^60^	*N* = 100 (*f* = 34; 61 alcoholics)	Alcoholism, anxiety reaction neurosis, personality trait disturbances	LSD (400–1500 μg)	Psychedelic therapy – the inpatient LSD session is the primary vehicle for personality change	One LSD session followed 2- days preparatory time, follow-up 3–18 months	None	Improvement in alcoholism and psychiatric conditions
Sherwood et al.^85^	*N* = 25 (*f* = 10)	Variety, including neurosis, alcoholism, marital problems	LSD (100–200 µg) with mescaline (200– 400 mg)	Psychedelic-assisted therapy with a focus on the subject’s intention and willingness to engage	Preparation time: 2–3 weeks, LSD/mescaline sessions over few months	None	Patient report, clinical assessment
Leary et al.^29^	*N* = 175	No specific disorder	Psilocybin (12–20 mg)	Group setting with informal discussions and Q&A sessions prior to the psychedelic session.	Not discussed	None	Impact of psychedelics experiences on pleasantness, learning, positive life changes, and interpersonal dimensions reported by patients and assessed by clinician.
Johnson^48^	*N* = 95	Alcoholism	LSD (300–500 µg)	Inpatient LSD session, and outpatient individual and group therapy sessions	One LSD session, follow-up of 12 months. Therapy included pre- and post-LSD interviews	Yes	Clinician assessment of LSD efficacy in facilitating positive transference and cementing patient-therapist rapport, and overall effect on personality modification
Mogar & Savag^12^	*N* = 70 (*f* = 27)	Primarily psychoneurotics and personality trait disturbances	LSD (200–300 µg) plus mescaline (200–400mg)	Preparation of weekly inhalations of 30% CO2 and 70% O2, then LSD/mescaline psychedelic therapy	1 month of preparation, then one LSD/mescaline session, follow-up evaluations 2 & 6 months post-LSD	Not clear	Measures of changes in personality Minnesota Multiphasic Personality Inventory (MMPI), value-belief Q-Sort, the Interpersonal Check List (ICL), and a semi structured Behavior Change Interview (BCI), and report of changes by patients
Stockings^14^	Age: 20–30	Effects of Mescaline which resembles psychotic states	Mescaline synthetic (200–500 mg)	No Therapy	Not applicable	None	Assessment of hallucinations, delusions, mood, thought patterns, motor disturbances, intellectual disturbance by patients report and clinician
Ditman et al.^13^	*N* = 99	Alcoholism	Three groups: 1. LSD (200 µg) 2. Methylphenidate (75 mg) 3. Chlordiazepoxide (75 mg)	Doctors and volunteers support the process. Orientation before the dosing for each group.	Single drug session	None	Prior and after the LSD session, patient reports on a 5-point scale on: 1. Pleasant emotion; 2. self-understanding; 3. Mystical feeling; 4. Parapsychological sensation; 5. Sensory and perceptual alternations; 6. Unpleasant emotion 7. Evaluation of the experience

Correspondingly, psychedelic treatment presented two distinct factors that necessitated careful clinical consideration. First, psychedelics appeared to induce a phenomenology that encompassed biological, psychological, interpersonal, and spiritual dimensions. Reported effects appeared dose-dependent and included sensory distortions, synesthesia, mystical-type experiences, intensification of affect, and increased suggestibility^9,20^. These effects were at once potentially destabilizing and therapeutic, compelling patients to confront disturbing and repressed content contributing to psychopathology^21,22^. Meaning-making capacities also appeared to intensify, with the memorable subjective effects produced by psychedelics appearing necessary to clinical gains^9,23^. Crucially, patients’ interpretation of psychedelics’ phenomenology seemed to highly depend on the nature and quality of the treatment’s non-pharmacological factors^24,25^. For example, music may be imbued with layers of significance and evoke transcendent wonder^21^. Correspondingly, the terms ‘set’ and ‘setting’ eventually evolved to describe the various psychological and environmental influences on psychedelic treatment^26,27^. ‘Setting’ refers to the clinical and environmental context in which the treatment occurs. The mind ‘set’ refers to the expectations, beliefs, and any pre-existing psychological factors (including psychopathology) the patient brings to the treatment session^28,29^.

The second factor during psychedelic treatment is the therapist’s unique role, which does not overlap with the conventional roles of psychiatrists and psychotherapists. In psychedelic treatment, researchers found, the clinician is compelled to work alongside and follow the patients’ responses to the psychedelic effects, promoting a sense of safety, rather than directing the patient toward insight^30^. Correspondingly, as psychedelics’ enhanced patients’ sensitivity to the staff, pre-prohibition clinicians discovered that the quality of their interpersonal skills impacted patients’ experience during the acute drug effects^31^. The necessity to mitigate potential psychological and behavioral risks, along with the need to support patients to reflect on their psychedelic experience to elicit therapeutic effects, led to the development of a common way of administrating the drug treatment. It included three phases: preparation, psychedelic treatment session, and integration, occurring after the drug administration session^30,32^.

Since 2006, clinical research with psychedelics has seen a revival after years of inactivity; recent studies indicate their potential efficacy in treating a range of severe psychiatric conditions, including treatment-resistant depression, anxiety disorders, and alcohol and substance use disorders^33,34^. In the renewed era of psychedelic research, trial protocols have upheld the imperative need for psychological care during the three phases^35^. Modern psychedelic research studies and their associated clinical frameworks are presented in Table 2. The chosen articles provide a representative sample of modern psychedelic research across indications, compounds, and therapeutic methodologies. In line with pre-prohibition research, contemporary studies highlight the significance of set and setting factors to clinical improvements^27^. Moreover, psychedelics may support therapeutic processes by temporarily relaxing rigid thinking patterns^36^, inducing non-dual awareness^37^, facilitating emotional breakthroughs^38^, and increasing psychological insight^39^. However, there is still a lack of sound empirical data supporting integrative models of care delineating specific ‘set and setting’ factors to optimize outcomes. Further, the therapist’s role and the benefits of integrating psychological tools during treatment remains understudied^1,40^.

**Table 2:** Summary literature of modern era

Study	Patient demographics	Treated disorder(s)	Psychedelic used/dosage	Therapy type	Treatment duration	Control group	Outcome measures
Sloshower et al.^86^	*n* = 19	MDD	Psilocybin	Two psilocybin sessions, preparation and integration session employing Acceptance and Commitment Therapy (ACT)	Preparation, psilocybin administration, and integration sessions – 6 weeks Follow up – 2 weeks	None	GRID-HAM-D-17, QIDS-SR-16 and suitability of ACT
Ross et al.^87^	*N* = 29 (*F* = 18)	Cancer patients with adjustment disorder (90%), or anxiety disorder (10%), based on SCID IV.	Psilocybin, (0.3 mg/kg), Niacin (250 mg)	One psilocybin session and one Niacin session (cross over design). Preparation and integration sessions therapy employed pro-prohibition style therapy focusing on existential /spiritual perspective.	Preparation, safety, psilocybin or Niacin administration, integration, and support sessions – 20 weeks	Yes	Primary outcomes: HADS, BDI, STAI Secondary outcomes: self-report on existential distress, life quality, spirituality, psilocybin’s persisting effects, mystical experience.
	Ages 22–75						
Johnson et al. ^88^	*N* = 15 (*F* = 5)	Nicotine dependence	Psilocybin (20–30 mg/70 kg)	Three psilocybin sessions (3rd dose optional). Preparation, integration, and support sessions are based on Cognitive Behavior Therapy (CBT)	Preparation, psilocybin administration integration and support sessions – 15 weeks Follow up - 6 months	None	Cigarette dependence: Breath CO, Urine cotinine, Questionnaire on Smoking Urges, Smoking Abstinence Self-Efficacy Scale, Wisconsin Smoking Withdrawal Scale Psilocybin effect: Visual Effects, Post-session Headache Interview, Mysticism Scale, States of Consciousness, Persisting Effects Questionnaire
	Age: 26–65						
Bogenschuz et al.^71^	*N* = 10 (*F* = 4)	Alcohol dependence	Psilocybin (0.3–0.4 mg/kg)	Two psilocybin sessions. Preparation, integration, and psychosocial sessions using Motivational Enhancement Therapy (MET)	Preparation, psilocybin administration, integration, and psychosocial sessions – 12 weeks	None	Alcohol Dependence: ARCI, Monitor Session Rating Form, TLFB, SIP, BAC, Stages of Change Readiness and Treatment Eagerness Scale, AASE, PACS Effect of Psilocybin: HRS, 5D-ASC, POMS
	Age: 25–65						
Stauffer et al.^89^	*N* = 18 (All males) mean age = 59.2)	AIDS survivors + demoralization and Attachment Anxiety or Attachment Avoidance	Psilocybin (0.3–0.36 mg/kg)	One psilocybin session, group preparatory and integrative sessions based on Brief Supportive Expressive Group Therapy (SEGT)	Preparation (individual and groups), psilocybin administration, integration (group) – 6 weeks Follow up - 3 months	None	Questionnaires about mystical Experience and challenging Experience
Carhart-Harris et al.^90^	*N* = 12 (*F* = 6)	TRD	Psilocybin session 1 (10 mg); psilocybin session 2 (25 mg)	Two psilocybin sessions preparation session, integration sessions based on non-directive support.	Preparation, psilocybin administration, and integration sessions – 2 weeks Follow up - 3 months	None	Patient-rated subjective intensity of psilocybin’s effects, QIDS, BDI, STAI-trait, SHAPS, HAMD, MADRS, fMRI
Doss et al.^91^	*N* = 24 (*F* = 16)	MDD	Psilocybin session 1 (20 mg/70 kg); psilocybin session 2 (30 mg/70 kg)	Two psilocybin sessions, randomization for immediate and delay treatments; preparation and integrations sessions based on non-directive support	Immediate and delayed treatments differed in onset time of treatment with the same duration of preparation, psilocybin administration, and integration sessions – 4 weeks	Yes	GRID-HAMD, Penn Conditional Exclusion Test, fMRI
	Age: 24–59						
Gasser et al.^92^	*N* = 10 (*F* = 4)	Metastasis disease cooccurring with mental health disorder: MDD, Dysthymia, Reactive Depression, Panic, General Anxiety, Social Phobia, and PTSD	LSD (200 µm) or active placebo (20 µm)	Two LSD sessions, preparation sessions, and integration sessions with client center approach	Preparation, LSD administration, and integration sessions – 9 weeks Follow up - 1 year	Yes	STAI, Qualitative semi-structured interviews to gain holistic understanding of client perspective
	Age: 39–64						
Goodwin et al.^68^	*N* = 233, (*F* = 121)	TRD	Psilocybin (10 mg or 25 mg) or active placebo (1 mg)	One psilocybin session, preparation and integration sessions consist of non-directive support	Preparation, psilocybin administration, and integration sessions – 6 weeks Follow up - 12 weeks	Yes	MADRS
Fontes et al.^93^	*N* = 29 (*F* = 21)	TRD	Ayahuasca (1 ml/kg) or placebo	One Ayahuasca session, assistance was offered as needed	1 week	Yes	MADRS, HAMD, CADSS, BPRS, MEQ-30
	Age: 18–60						
Mithoefer et al.^94^	*N* = 105 Age >18	PTSD	MDMA (75–125 mg) or active placebo (0–40 mg)	Two MDMA sessions, preparation and integration sessions, based on non-directive support to allow processing of traumatic memories and other interpersonal and behavioral experiences	Preparation, MDMA administration, and integration sessions – 6 weeks Follow up – 2 months and up to a 1 year	Yes,	CAPS-5 and BDI-II scores
Mitchell et al.^95^	*N* = 90 (*F* = 59)	PTSD	MDMA (80–180 mg) or placebo	Three MDMA sessions, preparation and integration sessions based on MDMA-assisted therapy	Preparation, MDMA administration, and integration sessions – 12 weeks Follow up – 6 weeks	Yes	CAPS-5 and SDS scores
Mitchell et al.^42^	*N* = 104 (*F* = 74) Age >18	PTSD	MDMA (120–180 mg) or placebo	Three MDMA sessions, preparation and integration sessions based on MDMA-assisted therapy	Preparation, MDMA administration, and integration sessions – 12 weeks Follow up – 6 weeks	Yes	CAPS-5 and SDS scores

*HADS* Hospital Anxiety and Depression Scale, *BDI* Beck Depression Inventory; *STAI* State Trait Anxiety Inventory, *TLFB* Timeline follow back, *ARCI* Addiction Research Center Inventory; *SIP* ShortInventory of Problems, *BAC* Breath Alcohol Concentration, *AASE* AlcoholAbstinence Self-Efficacy Scale; *PACS* Penn Alcohol Craving Scale, *HRS* HallucinogenRating Scale, *5D-ASC* 5-Dimensional Altered, State of Consciousness Scale, *POMS* Profile of Mood States, *QIDS* Quick Inventory of depressive symptomatology; *SHAPS* Snaith Hamilton, *HAMD* HamiltonDepressing Rating Scale, *MADRS* Montgomery Asberg Depression Rating Scale, *CADSS* Clinician-AdministeredDissociative States Scale, *BPRS* Brief Psychiatric Rating Scale; *MEQ* 30 Mystical experience scale, *CAPS-5* Clinician Administered PTSD Scale for DSM-5, *SDS* Sheehan Disability Scale.

As psilocybin in the treatment of depression^41^ and MDMA-assisted therapy for post-traumatic stress disorder (PTSD)^42^ are investigated in phase II and III studies and with their supervised use approved in Oregon and Australia^43,44^ there is a need for rigorous research to evaluate the impact and therapist effects and associated interventions. To that end, a review of the pre-prohibition period becomes crucial, as it originally inspired sophisticated reflections on the intricate relationship between biological and psychological factors in treating mental illness. Despite the limitations of that period, marked by poor controls, inconsistent diagnoses, unvalidated outcomes and lack of adverse effect reporting^3^, revisiting the two aforementioned fundamental factors can inform current and future research in this evolving field.

In this article, we examine selected pre-prohibition trials, explore clinician and patient narratives pertaining to the treatment process, and discuss their relevance to modern research. We gathered articles from the pre-prohibition era that showcase this period’s diverse perspectives and therapy methods. The chosen articles provide a clinically meaningful, representative sample of the essential aspects that defined psychedelic research and treatment during this period. Our aim is to trace the evolution of contemporary psychedelic treatment protocols, as observed in modern randomized clinical trials (RCTs). This includes an analysis of various components from the pre-prohibition era including clinician-reported psychedelic phenomenology, preparation, treatment sessions, and integration practices. The overarching objective is to contribute valuable insights to inform best practices and guide future research endeavors.

## Pre-prohibition clinician-reported psychedelic phenomenology

During the midcentury era, the use of psychedelics in psychiatry typically followed one of two models: the psycholytic and the psychedelic. In the psycholytic approach, low to moderate doses (e.g., LSD 25–200 µg) of psychedelics were used as an adjunct to conventional psychotherapy, aiming to facilitate access to unconscious material and to enhance the traditional psychodynamic treatment process^6^. The underlying assumption of the psycholytic approach was that therapeutic progress relies more on the psychodynamic process occurring during and between the non-drug psychotherapy sessions than on the experience induced by the psychedelic medication. In contrast, the psychedelic approach aimed to instigate therapeutic gains by eliciting intense transcendental and affective states through the administration of high doses of psychedelics (e.g., LSD > 200 µg), over 1–3 treatment sessions. Within the psychedelic approach, there were two methods of treatment: psychedelic chemotherapy^32^ and psychedelic-peak therapy^9,30^. In these approaches, therapeutic gains were directly attributed to the profound and cathartic impact of experiencing dramatic, unitive, and visionary states, with less emphasis on the core tenets of relationally driven forms of person-centered counseling or psychoanalytic psychotherapy.

The difference in the dose quantity between these two psychedelic models mirrors their distinct approaches to facilitating positive psychological shifts. In the psycholytic model, mental changes were anticipated to unfold more subtly yet with the potential for meaningful impact, as the biochemical psychedelic effects aid insight-oriented psychoanalysis. Whereas the psychedelic approach relied on intense and immersive psychedelic states that elicit profound and disruptive mystical-type experiences, often accompanied by emotional breakthroughs, shifts in belief systems and self-narratives, serving as the primary catalysts for change. We now describe the manifestation of the states of consciousness in each model using a range of selected researcher narratives from pre-prohibition trials.

## Clinician-reported phenomenology in psycholytic therapy

Pre-prohibition researchers aligned with the psycholytic model^9,24,45,46^ extensively detailed the physiological and psychological effects of LSD. The description underscored LSD’s characteristic outcomes, including heightened distortion of perception and enhanced emotion, as well as the elicitation of introspection and the unveiling of the individual’s unconscious during the drug treatment session. While toxicity from small-moderate doses of LSD were rare, reported adverse effects included nausea, dizziness, sensations of heat/cold, and sweating. Despite the drug’s intended goal of aiding psychological treatment, specific psychological effects, often linked to the patient’s characteristics, at times proved counterproductive for psychoanalytic psychotherapy. For example, in psycholytic sessions, the influence of LSD could exacerbate pre-existing neurotic symptoms, heightening anxiety and causing bodily trembling^46^. During these instances, some patients craved human contact, while some withdrew and became increasingly unreachable, potentially undermining the therapeutic process^24^. Although ego functions largely persisted, allowing for the internal registration of the psychedelic experience, patients oscillated between mentally exploring immersive inner realms and experiencing moments of uncontrollable psychic overwhelm as ego barriers dissolved^46^. However, often enough, three main phenomena occurred during the most potent moments of the drug action, shaping the therapeutic session and potentially contributing to improving disease symptoms:

- Non-specific hallucinations: Psychedelics induce sensory experiences, such as visions of animals, people, and shapes, even in the absence of external stimuli. Patients may withdraw into timeless worlds with ancient atmospheres, alternating between immersive hallucinatory periods where time ceases and reality breaks down, occasionally accompanied by moments of realization that their mind had constructed an unreal realm^11,24^. Some patients reported assigning roles to those present in the treatment room based on the emerging inner drama, which were classified into two major types: Identification and Projection^24^. Identifying with the emergent phenomenon appeared to help bring resolution to internal conflicts. During the Projection processes, people and places in the immediate surroundings could represent the patient’s unsolved developmental and oedipal conflicts.
- Reliving repressed memories: Repressed memories spanning the patient’s lifespan could surface, but some believed that LSD enabled the vivid recall of childhood traumatic memories, allowing the patient to re-experience their body and affective state at the time of traumatization^25^. Therefore, psycholytic treatment sessions could elicit potent recollections of childhood trauma, providing full sensory immersion into the traumatized self-identity.
- Impersonal archetypal unconscious imagery: Profound emotions can accompany vivid unconscious imagery and symbols emerging during psychedelic states. These included typical primal patterns and universal themes (e.g., ancient landscapes, religious iconography, visions of the natural world and the cosmos) that resonated across cultures^11,22^.

Based on this phenomenology, psycholytic researchers considered psychedelics’ primary function as an adjunct to on-going psychotherapy, asserting, “the reaction which was desired was to make the patient more accessible but was not of such intensity as to create hallucinations”^47^. In turn, psycholytic treatment aimed to increase patient openness to therapist interpretations, mobilize the patient’s ego functions and strengths, enable rapid insight, and facilitate confrontation with repressed traumatic events through concurrent psychoanalytical psychotherapy^11,48^. As the primary mechanism of change in the psycholytic model focused on facilitating the transference, intense, psychedelic-induced subjective experiences, such as ego dissolution, disembodiment, and a sense of unity with nature and humanity^49^, at times, defied the more traditional psychoanalytic, neutral and interpretative clinical stance.

## Clinician-reported phenomenology in psychedelic ‘peak’ therapy

In modern-day research, extensive literature has documented alterations in consciousness during intense psychedelic treatment sessions^50,51^. Lacking sophisticated neurobiological research tools and methods, pre-prohibition researchers relied on patient reports and clinical observations to understand the medical psychedelic experience^21^. For example, Alnæs^30^ reported observations of 20 patients and 20 volunteers who were administered high doses (<250 mcg LSD) during treatment. Focusing on the profound psychedelic effects, Alnaes reported three phases:

The initial phase involved various physical experiences leading to so-called ego death and dissolution - sensations of cold clamminess, bodily pressure, trembling, nausea, and vibrations were reported. At the precipice of ego death, subjects reported perceiving a clear light, aligning with descriptions in Tibetan literature of the process of dying^29^. The second phase included immersive visions and mythic content. These included feelings of mind/body separation, visions filled with light, and encounters with mythic heroes/demons. Further, symbolic birth themes were reported in psychedelic sessions, featuring canyon-like passages^52,53^. The third phase included re-orientation and integration of insights. At this stage, guided self-insight, in which patients were supported to reach their own conclusions about their experience, appeared invaluable^48^. During this phase, suggestibility and memory increased, and repressed material arose. More specifically, patients were more receptive to novel ideas, environmental cues, and divergent thinking, with their entrenched worldviews becoming more malleable to change^9,11,30,32^.

To conclude, during the pre-prohibition era, methodological questions concerning the nature, timing, and focus of clinical interventions aimed at leveraging the potential therapeutic effects associated with psychedelic peak experiences were raised. In contemporary research, largely concerned with establishing an evidence base for the safety and efficacy of psychedelics, these questions have yet to be the focus of rigorous empirical studies. Noting that some patients suffered adverse events and struggled to leverage revelatory shifts without ongoing care, pre-prohibition clinicians explored, and modern-day researchers are asking, what, if any, type of psychological interventions are optimal during and following psychedelic treatments that may dissolve ego structures and abruptly overhaul rigidly held worldviews.

In the next section, we will discuss the paradigm of psychedelic treatment: preparation -> treatment session -> integration as it was created at the time and the therapist’s role in each phase.

## Therapist’s role: preparation, psychedelic treatment, and integration sessions

In the 1950s and 1960s, researchers recognized the complex interactivity between psychedelics’ pharmacological properties, patients’ intrapsychic dispositions, and the treatment’s non-pharmacological factors^3^. By 1965 three broad treatment methods based on dosage and therapy techniques existed:

1. “Psycholytic” protocols that involved multiple low to moderate doses (25–200 μg) and psychodynamic psychotherapy interventions to promote gradual personality changes.
2. “Psychedelic chemotherapy” sessions that involved a single high dose of LSD (200+ μg) in medical settings with limited psychological interventions, prioritizing the patient’s physical safety and relying on the acute effects of the drug.
3. “Psychedelic Peak Therapy” which consolidated intentional patient preparation, empathetic support during heightened suggestible ego-dissolved states, and integration processes after the psychedelic treatment session to promote enduring psychological and spiritual transformations regarding self-concept, relationships, and meaning.

In this section, we will explore how psychedelic chemotherapy evolved into psychedelic-peak therapy, using the three-part method of psychedelic treatment: preparation, treatment session, and integration. Additionally, we will explore how psycholytic therapy integrated itself into this treatment method.

## Preparation

The current framework for the administration of psychedelics in research settings was developed to address the challenges that were identified during early research. The challenges were exemplified in Smith’s^32,54^ report on using LSD or mescaline in psychedelic chemotherapy for patients with alcohol use disorder (AUD).

In Smith’s protocol, patients were initially admitted to a 1-week evaluation and professional rapport-building in a psychiatric ward. Smith stressed retroactively the importance of introducing encouragement, enticement, and directive guidance prior to drug administration to prime patients to benefit from the drug treatment. Distinguishing the psychedelic approach from psycholytic models, Smith also asserted that there less need to provide insights (or psychoanalytic interpretations) to patients as many seemed to have gained a better understanding of themselves due to the psychedelic experience. Further, if patients perceived their therapists as distant or disinterested, they tended to be more anxious or paranoid, therefore having difficulties immersing in the experience. Similarly, Smart et al^55^. who treated 30 AUD patients with a long history of uncontrolled drinking, noted “the role of the therapists’ conviction and personal commitment to a treatment approach has rarely been investigated as a factor in success but it might well be important”. Acknowledging the therapists’ role in supporting treatment success via establishing trust and safety, Smart also provided patients with information concerning the drug effects during the preparation phase.

Drawing from insights gained from the psychedelic chemotherapy modality, the psychedelic-peak approach developed a clinically meaningful understanding of the non-pharmacological factors influencing patient outcomes. Researchers adjusted the treatment environment, staff training, and instituted guidelines regarding interpersonal interactions with patients^21,30^. Over time, researchers discerned that effective preparation played a pivotal role in guiding patients through inner explorations by employing diverse approaches such as aesthetic appreciation, the area of philosophy that studies the nature of beauty and art^30,56^. Alnaes conducted preparation sessions based on principles ‘The Tibetan Book of the Dead’^29^. In this approach, the same preparation used by the Tibetans before death served as an informative introduction in preparation for psychedelic experiences. Further, Abramson detailed additional preparation activities, such as establishing trust between the patient and therapist, conducting group preparation sessions, and employing techniques such as picture imagination, in which therapists helped patients focus on mental images in order to evoke feelings of relaxation^57^. Further, autogenic training, a therapeutic technique designed to help patients achieve a calm state by focusing on specific sensations and imagery or occasionally hypnosis with posthypnotic suggestions, was employed.

Accordingly, the primary goal of the preparation phase was to support patients to accept the drug’s effects without fear, emphasizing a readiness to experience the expected and unexpected somatic and psychic effects. Importantly, in cases lacking adequate preparation, researchers observed that most patients struggled counterproductively, experiencing increasing dysregulation, avoidance, and despair during the psychedelic treatment session^7,9,30^. Conversely, patients who were more amenable to temporarily relinquish their familiar ego functions and accept the experience appeared more likely to achieve positive outcomes^9,24,32^.

## Psychedelic treatment session

Due to the high dosage and its often dramatic effects, psychodynamic interventions were not feasible or advisable during drug administration^30^. However, it soon became evident that patients required specific forms of support during the acute drug effects. Staff members with LSD experiences or those frequently observing LSD sessions were thought to be better suited to support patients during psychedelic treatment^32^. Researchers also observed that personnel maintaining neutrality during the acute phase of the drug effects were more likely to provoke fear and hostility in patients^55^. Correspondingly, Parley described how nurses carefully prepared patients for their LSD treatment, providing reassurance and guidance throughout the treatment session, and helping patients process their emotions and insights afterwards^58^. She emphasized the importance of the nurse-patient relationship and the need for empathy, intuition, and creativity in this unique therapeutic context. Far from being mere observers, these nurses acted as companions, guides, and anchors for patients receiving psychedelic treatment, providing reassurance, support, and understanding throughout the drug administration session. Further, by 1958, psychedelic treatment clinics began emphasizing the importance of “set and setting” in achieving desired outcomes^9,24,30,59^. Auditory stimuli, such as relaxing classical and semi-classical music, enhanced perception and diverted attention from self-focused fears induced by the experience^21^. Visual stimuli, like artwork or personal photographs prompted emotional reflection on unhealthy attitudes and relational dynamics, contributing to post-session reflection^46^. Lastly, it became apparent that the patient-reported phenomenology often entailed encounters with psychospiritual states that diverged from traditional psychiatric and psychoanalytic frameworks.

Correspondingly, to accommodate the distinct phenomenology of the psychedelic state, researchers administering psychedelic-peak treatment sought additional frameworks that expanded on traditional psychoanalytic practices^18^. For instance, Chwelos et al.^21^ encouraged patients to accept themselves without rationalizations, meaning-making, or guilt over emerging material during peak emotional periods. Similarly, Chandler asserted, “It is best to let the fantasy unfold its meaning by encouraging the patient to go along with it and see it through to the end.”^11^ This approach emphasized the utility of patients’ courage, willingness to surrender to the drug effects, self-acceptance, and responsibility for change^59^. Following psychedelic treatment sessions, many patients engaged in spiritual or religious discussions exploring the spiritual implications of their experiences^22,30,60^.

These discussions highlighted the contrast between biological and psychoanalytic conceptualizations of pathology and mystical-type experiences, there by redirecting the clinical focus to interventions concerned with leveraging transpersonal states for therapeutic gains^21^. Correspondingly, discussing treatment responders, Pahnke stated, “If the psychedelic peak experience is achieved and stabilized, the clinical picture can be described as follows: Mood is elevated and energetic; there is a relative freedom from concerns of the past, from guilt and anxiety, and the disposition and capacity to enter close interpersonal relationships is enhanced”^61^. However, the acute and potentially highly distressing treatment effects were noted across studies. Coupled with experimental and unsubstantiated interventions delivered to treatment-resistant and complex patient populations, including schizophrenia, adverse events were under reported, however, certainly present and many patients did not improve^62,63^.

## Integration

Once the psychedelic effects subsided, patients undergoing psychedelic chemotherapy were transferred to regular hospital beds where they spent the night^32,55^. If necessary, patients were given anxiolytics and released to the clinic the next day^55^ or referred to primary care, including alcoholics anonymous groups^9^. During regular individual or group meetings, patients were free to discuss any insights associated with their treatment sessions, however, therapists did not direct patients to examine their experiences unless they initiated it themselves^9,30^. As for the patients undergoing psychedelic peak therapy, the impact of psychedelic experiences was thought to extend beyond the treatment sessions, compelling the integration of events spanning from the psychedelic treatment session to the present reality^64^. As Maclean noted, patients “may become aware of those archetypal or universal meanings which underlie all human feeling and thinking^60^. The symbols provide intermediate points of reference creating a bridge between the habitual self-concept and a new concept based on self-understanding and self-acceptance.” Correspondingly, to promote psychological safety and effectiveness, in the days and weeks after psychedelic treatment sessions, clinicians recognized that patients required structured procedures. These included interviews with clinicians, peer-to-peer discussions, artistic expression, and guiding patients to write accounts of their experiences^30,31,46,60^.

## Discussion

This narrative review highlights the wealth of insights garnered from the utilization of psychedelics in the treatment of mental disorders during the pre-prohibition era. While acknowledging that pre-prohibition research methodologies do not align with contemporary randomized control trial (RCTs) standards, the valuable clinical observations made by researchers in that era, benefiting from freedom and flexibility in drug access and administration, informed modern clinical trials and could inform future investigations and care models. For example, modern researchers may draw inspiration from the pre-prohibition era to help optimize the treatments’ non-pharmacological factors. This may include experimenting with different treatment approaches (e.g., individual vs. group-based treatment protocols) and the investigation of more flexible psychotherapeutic techniques before and after the psychedelic treatment session. Further, pre-prohibition researchers produced rich case reports describing their observations of patients undergoing psychedelic treatment. Despite changes to theoretical conceptualizations of mental ill health and advances in evidence-based clinical technique, modern researchers and clinicians may gain insight into the potential complexities of the treatment process by reviewing these reports. In turn, this may promote better safety outcomes and contribute to the design of best practice and ethical guidelines. Further, by combining the insights from pre-prohibition studies with the methodological rigor of modern RCTs, researchers can develop a comprehensive, evidence-based approach to psychedelic forms of therapy. This synthesis may lead to innovative treatment models that are tailored to individual needs while maintaining scientific integrity.

The profound effects reported by pre-prohibition patients undergoing psychedelic treatment coupled with heightened patient suggestibility and regression potential^65^, compelled clinicians to organically devise specialized protocols for administrating psychedelics. Regardless of the chosen treatment model (e.g., psycholytic or psychedelic), practitioners converged on common core principles for treatment^66^. Clinicians understood that adequate preparation encompassing psychoeducation, establishing patient-therapist rapport and clearly defining the role of therapists was crucial. Providing a comfortable physical setting and an accepting and encouraging interpersonal atmosphere were essential. Further, clinicians came to appreciate patients’ need to intentionally reflect on their experience and develop a clinically meaningful narrative of their subjective experience. Similarly, the standard modern psychedelic RCT model of preparation – > treatment -> integration was established. Further, in the psychedelic model, clinicians moved from a largely interpretive clinical position to a more explicitly supportive stance; implicitly suggesting that the patient’s internal resources, rather than therapist’s theoretical perspectives, are the primary driver of positive change. In the psycholytic approach, psychoanalytically oriented clinicians, primarily focused on psychodynamic explorations of the patients’ unconscious via the transference/countertransference phenomena, expanded their clinical repertoire to accommodate patient reports of psychospiritual states associated with the treatment^18,67^.

Modern psychedelic RCTs are designed to support regulatory approval of the compound and, therefore, are focused on generating replicable and generalizable safety and efficacy data. Further, in a medication RCT, all non-drug factors must be standardized and held fixed to isolate the drug effects. Correspondingly, to reduce treatment variables, standardized models of psychological support^68,69^ or psychotherapy^70–72^ have been implemented across trials. This necessary approach is appropriate for the developmental stage of psychedelic treatments in mental health, which is reaching the milestone of acceptance and adoption within medicine. Alongside this process, literature is emerging regarding the type of psychological interventions that might be safe and effective in psychedelic treatment^73–75^. Further, integrative psychotherapeutic approaches, incorporating interpretations of findings from empirical research, have also been suggested^76^. Yet sound empirical data that will inform the utility of these approaches is currently limited. To that end, a mature and evidence-based form of psychotherapy utilizing psychedelics, which would require specific research, could turn to pre-prohibition trials for guidance and inspiration.

Reviewing the plethora of convergent and divergent perspectives of pre-prohibition clinicians, who were not limited by the legality of psychedelics in clinical practice, suggests that if certain psychedelics were to receive regulatory approval, a new era of rigorous research, focused on adjunct psychological interventions, therapist effects and modulation of the treatment’s non-pharmacological factors will be feasible, warranted and needed to promote positive and durable patient outcomes. Pre-prohibition perspectives could help design studies investigating the relevance, selection, and significance of psychological interventions in psychedelic treatment. Additionally, the clinical richness of pre-prohibition perspectives could inform studies assessing the impact of therapist effects, guiding the development of evidence-based training and supervision protocols to optimize clinical outcomes.

Further, pre-prohibition treatment was administered in both inpatient and outpatient settings, in individual or group-based treatment protocols and consisting of single to multiple psychedelic treatment sessions. Clinician and patient testimonials, and pre-prohibition treatment protocols may serve as valuable guidance for designing future studies, delineating the roles of various staff members, defining the treatment environment and procedures, and determining treatment frequency. For example, studies generating data pertaining to the effects of different compounds, under specific clinical conditions, in various patient populations, utilizing more sophisticated psychological assessments, could inform whether and how psychological interventions should be tailored to enhance patient outcomes.

Correspondingly, contemporary research utilizes psychometric measures, such as the Altered States of Consciousness Questionnaire^77^, to assess peak states. While this detailed exploration provides valuable insights, using additional research methodologies could increase the ability to discover novel perspectives associated with psychedelic treatment. The rich descriptions of the pre-prohibition era allowed clinicians to observe more closely patients’ subjective experiences, informing potential interventions. In modern trials, investigating patients’ experiences through qualitative research methodologies and natural language processing may offer a more nuanced understanding of peak states and optimize research and treatment protocols. Importantly, novel measures that are based on clinical observations assessing patients’ readiness for psychedelic treatment^78^ and follow-up assessments capturing biopsychosocial changes and integration processes^79^ may optimize screening, preparation, and post-treatment care where indicated.

Further, during the pre-prohibition era, the effects of psychedelics were primarily characterized in terms of psychological changes, especially with respect to the dramatic alterations in consciousness. Today, with advancements in neuroimaging and molecular biology, we have modern tools that can aid in understanding the neurobiological alterations in the nervous system underlying the observed psychological phenomena^80,81^. Correspondingly, this may support the development of a more comprehensive, biopsychosocial understanding of mental ill health and approach to psychedelic treatment, enabling the identification of biomarkers informing patient suitability for treatment and predictors of response to inform precision treatment planning.

In summary, clinician and patient-reported qualitative narratives of psychological processes of change were predominant in pre-prohibition research, playing a crucial role in the evolution of psychedelic research and treatment. However, in the modern era, the emphasis has shifted, and evidence-based research now heavily relies on psychometric measures and symptom assessments. Whether investigating psychedelics as a standalone treatment with psychological support^82^ or considering their application as an adjunct to psychotherapy^75,83^, it is essential to recognize that the nuanced understanding and rich clinical narratives derived from pre-prohibition data can significantly contribute to the design and implementation of future investigations. This, in turn, holds the potential to advance contemporary psychedelic treatment and care paradigms.

## References

[1] Greenway, K. T., Garel, N., Jerome, L. & Feduccia, A. A. Integrating psychotherapy and psychopharmacology: psychedelic-assisted psychotherapy and other combined treatments. *Expert Rev. Clin. Pharmacol.* **13**, 655–670 (2020). 32478631 10.1080/17512433.2020.1772054

[2] Hofmann, A. *LSD: My Problem Child* (Oxford University Press, 2013).

[3] Rucker, J. J. H., Iliff, J. & Nutt, D. J. Psychiatry & the psychedelic drugs. Past, present & future. *Neuropharmacology* **142**, 200–218 (2018). 29284138 10.1016/j.neuropharm.2017.12.040

[4] Rucker, J. J. & Seth, P. Psychedelics: old drugs, new trips. *J. Psychopharmacol.* **35**, 316–318 (2021). 33853423 10.1177/02698811211003495

[5] Cavarra, M., Falzone, A., Ramaekers, J. G., Kuypers, K. P. C. & Mento, C. Psychedelic-assisted psychotherapy—a systematic review of associated psychological interventions. *Front. Psychol.* **13**, 887255 (2022). 35756295 10.3389/fpsyg.2022.887255 PMC9226617

[6] Passie, T., Guss, J. & Krähenmann, R. Lower-dose psycholytic therapy - a neglected approach. *Front. Psychiatry* **13**, 1020505 (2022). 36532196 10.3389/fpsyt.2022.1020505 PMC9755513

[7] Abramson, H. A. Lysergic acid diethylamide (LSD-25): XIX. As an adjunct to brief psychotherapy, with special reference to ego enhancement. *J. Psychol.* **41**, 199–229 (1956).

[8] Rucker, J. J., Jelen, L. A., Flynn, S., Frowde, K. D. & Young, A. H. Psychedelics in the treatment of unipolar mood disorders: a systematic review. *J. Psychopharmacol.* **30**, 1220–1229 (2016). 27856684 10.1177/0269881116679368

[9] Lewis, D. J. & Sloane, R. B. Therapy with lysergic acid diethylamide. *J. Clin. Exp. Psychopathol.* **19**, 19–31 (1958). 13525450

[10] Jensen, S. E. A treatment program for alcoholics in a mental hospital. *Q. J. Stud. Alcohol* **23**, 315–320 (1962). 14451676

[11] Chandler, A. L. & Hartman, M. A. Lysergic acid diethylamide (LSD-25) as a facilitating agent in psychotherapy. *AMA Arch. Gen. Psychiatry* **2**, 286–299 (1960).

[12] Mogar, R. E. & Savage, C. Personality change associated with psychedelic (LSD) therapy: a preliminary report. *Psychother. Theory Res. Pract.* **1**, 154 (1964).

[13] Ditman, K. S. et al. Dimensions of the LSD, methylphenidate and chlordiazepoxide experiences. *Psychopharmacologia* **14**, 1–11 (1969). 4900730 10.1007/BF00401528

[14] Stockings, G. T. A clinical study of the mescaline psychosis, with special reference to the mechanism of the genesis of schizophrenic and other psychotic states. *J. Ment. Sci.* **86**, 29–47 (1940).

[15] Waltz, M. *Autism: A Social and Medical History.* p. 83-98 (Springer, 2023).

[16] Griffiths, R. R. et al. Psilocybin occasioned mystical-type experiences: immediate and persisting dose-related effects. *Psychopharmacology* **218**, 649–665 (2011). 21674151 10.1007/s00213-011-2358-5 PMC3308357

[17] Nichols, D. E. Psychedelics. *Pharmacol. Rev.* **68**, 264–355 (2016). 26841800 10.1124/pr.115.011478 PMC4813425

[18] Grof, S. *Address to the Conference of the European Association for Psycholytic Therapy* (1969).

[19] Aaronson, B., and Osmond, H. *Psychedelics: The Uses And Implications Of Hallucinogenic Drugs* (Doubleday, 1970).

[20] Barral, M. R. The varieties of psychedelic experience. *Int. Philos. Q.* **7**, 677–680 (1967).

[21] Chwelos, N., Blewett, D. B., Smith, C. M. & Hoffer, A. Use of d-lysergic acid diethylamide in the treatment of alcoholism. *Q. J. Stud. Alcohol* **20**, 577–590 (1959). 13810249

[22] Sherwood, J. N., Stolaroff, M. J. & Harman, W. W. The psychedelic experience-a new concept in psychotherapy. *J. Psychedelic Drugs* **1**, 96–111 (1968). 13977209

[23] Eisner, B. Set, setting, and matrix. *J. Psychoactive Drugs* **29**, 213–216 (1997). 9250949 10.1080/02791072.1997.10400190

[24] Sandison, R. A. Psychological aspects of the LSD treatment of the neuroses. *J. Ment. Sci.* **100**, 508–515 (1954). 13175012 10.1192/bjp.100.419.508

[25] Sandison, R. A., Spencer, A. M. & Whitelaw, J. D. A. The therapeutic value of lysergic acid diethylamide in mental illness. *J. Ment. Sci.* **100**, 491–507 (1954). 13175011 10.1192/bjp.100.419.491

[26] Hartogsohn, I. Set and setting, psychedelics and the placebo response: an extra-pharmacological perspective on psychopharmacology. *J. Psychopharmacol.* **30**, 1259–1267 (2016). 27852960 10.1177/0269881116677852

[27] Carhart-Harris, R. et al. Psilocybin with psychological support for treatment-resistant depression: six-month follow-up. *Psychopharmacology* **235**, 399–408 (2018). 29119217 10.1007/s00213-017-4771-x PMC5813086

[28] Hartogsohn, I. Constructing drug effects: a history of set and setting. *Drug Sci. Policy Law* **3**, 2050324516683325 (2017).

[29] Leary, T., Litwin, G. H. & Metzner, R. Reactions to psilocybjn administered in a supportive environment. *J. Nerv. Ment. Dis.* **137**, 561–573 (1963). 14087676 10.1097/00005053-196312000-00007

[30] Alnæs, R. THERAPEUTIC APPLICATION OF THE CHANGE IN CONSCIOUSNESS PRODUCED RY PSYCHOLYTICA (LSD, PSILOCYRIN, ETC.) 1: the psychedelic experience in the treatment of neurosis. *Acta Psychiatr. Scand.* **39**, 397–409 (1964). 14345225 10.1111/j.1600-0447.1964.tb04952.x

[31] Whitaker, L. H. Lysergic acid diethylamide in psychotherapy. Part I: clinical aspects. *Med. J. Aust.* **1**, 5–8 (1964). 14116718

[32] Smith, C. M. A new adjunct to the treatment of alcoholism: the hallucinogenic drugs. *Q. J. Stud. Alcohol* **19**, 406–417 (1958). 13579169

[33] Yaden, D. B., Yaden, M. E. & Griffiths, R. R. Psychedelics in psychiatry-keeping the renaissance from going off the rails. *JAMA Psychiatry* **78**, 469–470 (2021). 33263720 10.1001/jamapsychiatry.2020.3672 PMC8102315

[34] Mitchell, J. M. & Anderson, B. T. Psychedelic therapies reconsidered: compounds, clinical indications, and cautious optimism. *Neuropsychopharmacology* **49**, 96–103 (2024). 37479859 10.1038/s41386-023-01656-7 PMC10700471

[35] Modlin, N. L., Stubley, J., Maggio, C. & Rucker, J. J. On redescribing the indescribable: trauma, psychoanalysis and psychedelic therapy. *Br. J. Psychother.* **39**, 551–572 (2023).

[36] Carhart-Harris, R. L. How do psychedelics work. *Curr. Opin. Psychiatry* **32**, 16–21 (2019). 30394903 10.1097/YCO.0000000000000467

[37] Nayak, S. M., Singh, M., Yaden, D. B. & Griffiths, R. R. Belief changes associated with psychedelic use. *J. Psychopharmacol.* **37**, 80–92 (2023). 36317643 10.1177/02698811221131989

[38] Roseman, L. et al. Emotional breakthrough and psychedelics: validation of the emotional breakthrough inventory. *J. Psychopharmacol.* **33**, 1076–1087 (2019). 31294673 10.1177/0269881119855974

[39] Peill, J. M. et al. Validation of the Psychological Insight Scale: a new scale to assess psychological insight following a psychedelic experience. *J. Psychopharmacol.* **36**, 31–45 (2022). 34983255 10.1177/02698811211066709 PMC8801624

[40] Brennan, W., Kelman, A. R. & Belser, A. B. A systematic review of reporting practices in psychedelic clinical trials: psychological support, therapy, and psychosocial interventions. *Psychedelic Med.* **1**, 218–229 (2023). 10.1089/psymed.2023.0007 PMC11658666 40046864

[41] Ko, K., Kopra, E. I., Cleare, A. J. & Rucker, J. J. Psychedelic therapy for depressive symptoms: a systematic review and meta-analysis. *J. Affect Disord.* **322**, 194–204 (2023). 36209780 10.1016/j.jad.2022.09.168

[42] Mitchell, J. M. et al. MDMA-assisted therapy for moderate to severe PTSD: a randomized, placebo-controlled phase 3 trial. *Nat. Med.* **29**, 2473–2480 (2023). 37709999 10.1038/s41591-023-02565-4 PMC10579091

[43] van Elk, M. & Fried, E. I. History repeating: guidelines to address common problems in psychedelic science. *Ther. Adv. Psychopharmacol.* **13**, 20451253231198466 (2023). 37766730 10.1177/20451253231198466 PMC10521293

[44] Kisely, S. The down-scheduling of MDMA and psilocybin(e): too fast and too soon. *Aust. N. Z. J. Psychiatry* **57**, 933–934 (2023). 37161273 10.1177/00048674231174171 PMC10291367

[45] Busch, A. K. & Johnson, W. C. LSD 25 as an aid in psychotherapy; preliminary report of a new drug. *Dis. Nerv. Syst.* **11**, 241–243 (1950). 14793387

[46] Eisner, B. G. & Cohen, S. Psychotherapy with lysergic acid diethylamide. *J. Nerv. Ment. Dis.* **127**, 528–539 (1958). 13621221 10.1097/00005053-195812000-00006

[47] Rolo, A., Krinsky, L. W. & Goldfarb, L. LSD as an adjunct to psychotherapy with alcoholics. *J. Psychol.* **50**, 85–104 (1960).

[48] Johnson, F. G. LSD in the treatment of alcoholism. *Am. J. Psychiatry* **126**, 481–487 (1969). 5806791 10.1176/ajp.126.4.481

[49] Pahnke, W. N. & Richards, W. A. Implications of LSD and experimental mysticism. *J. Relig. Health* **5**, 175–208 (1966). 24424798 10.1007/BF01532646

[50] Preller, K. H. & Vollenweider, F. X. Phenomenology, structure, and dynamic of psychedelic states. *Curr. Top. Behav. Neurosci.* **36**, 221–256 (2018). 28025814 10.1007/7854_2016_459

[51] Graziosi, M., Singh, M., Nayak, S. M. & Yaden, D. B. Acute subjective effects of psychedelics within and beyond WEIRD contexts. *J. Psychoact. Drugs* **55**, 558–569 (2023). 10.1080/02791072.2023.2255274 37679890

[52] Osmond, H. A review of the clinical effects of psychotomimetic agents. *Ann. N. Y. Acad. Sci.* **66**, 418–434 (1957). 13425232 10.1111/j.1749-6632.1957.tb40738.x

[53] Stanislav, G. *Beyond the Brain: Birth, Death, and Transcendence in Psychotherapy* (State University of New York Press, 1985).

[54] Smith, C. M. Some reflections on the possible therapeutic effects of the hallucinogens. With special reference to alcoholism. *Q. J. Stud. Alcohol* **20**, 292–301 (1959). 13668045

[55] Smart, R. G., Storm, T., Baker, E. F. W. & Solursh, L. A controlled study of lysergide in the treatment of alcoholism. I. The effects on drinking behavior. *Q. J. Stud. Alcohol* **27**, 469–482 (1966). 5970697

[56] Bishop, M. G. *The Discovery Of Love: A Psychedelic Experience With LSD-25* (Torquil, 1963).

[57] Abramson, H. A. LSD in psychotherapy and alcoholism. *Am. J. Psychother.* **20**, 415–438 (1966). 5337647 10.1176/appi.psychotherapy.1966.20.3.415

[58] Parley, K. A. Y. Supporting the patient. *AJN Am. J. Nurs.* **64**, 80–82 (1964). 14123390

[59] Kast, E. Attenuation of anticipation: a therapeutic use of lysergic acid diethylamide. *Psychiatr. Q.* **41**, 646–657 (1967). 4169685 10.1007/BF01575629

[60] MacLean, J. R., MacDonald, D. C., Byrne, U. P. & Hubbard, A. M. The use of LSD-25 in the treatment of alcoholism and other psychiatric problems. *Q. J. Stud. Alcohol* **22**, 34–45 (1961). 13764952

[61] Pahnke, W. N., Kurland, A. A., Goodman, L. E. & Richards, W. A. LSD-assisted psychotherapy with terminal cancer patients. *Curr. Psychiatr. Ther.* **9**, 144–152 (1969). 5348915

[62] Cohen, S. Lysergic acid diethylamide: side effects and complications. *J. Nerv. Ment. Dis.* **130**, 30–40 (1960). 13811003 10.1097/00005053-196001000-00005

[63] Johnstad, P. G. A dangerous method? Psychedelic therapy at Modum Bad, Norway, 1961-76. *Hist. Psychiatry* **31**, 217–226 (2020). 31928087 10.1177/0957154X19894537

[64] Richards, W. A. Psychedelic psychotherapy: insights from 25 years of research. *J. Humanist. Psychol.* **57**, 323–337 (2017).

[65] Maggio, C., Fischer, F., Modlin, N. & Rucker, J. Psychoanalytic formulations in psychedelic therapy for Treatment Resistant Depression (TRD). *J. Psychol. Psychother.* **13**, 451 (2023).

[66] Johnson, M. W., Richards, W. A. & Griffiths, R. R. Human hallucinogen research: guidelines for safety. *J. Psychopharmacol.* **22**, 603–620 (2008). 18593734 10.1177/0269881108093587 PMC3056407

[67] Grof, C. & Grof, S. Spiritual emergency: the understanding and treatment of transpersonal crises. *Int. J. Transpers. Stud.* **36**, 30–43 (2017).

[68] Goodwin, G. M. et al. Single-dose psilocybin for a treatment-resistant episode of major depression. *N. Engl. J. Med.* **387**, 1637–1648 (2022). 36322843 10.1056/NEJMoa2206443

[69] Raison, C. L. et al. Single-dose psilocybin treatment for major depressive disorder: a randomized clinical trial. *JAMA* **330**, 843–853 (2023). 37651119 10.1001/jama.2023.14530 PMC10472268

[70] Mithoefer, M. C. *A Manual for MDMA-Assisted Psychotherapy in the Treatment of Posttraumatic Stress Disorder* https://maps.org/mdma/mdma-resources/treatment-manual-mdma-assisted-psychotherapy-for-ptsd/ (2022). 10.1002/jts.22696 PMC8453707 34114250

[71] Bogenschutz, M. P. et al. Psilocybin-assisted treatment for alcohol dependence: a proof-of-concept study. *J. Psychopharmacol.* **29**, 289–299 (2015). 25586396 10.1177/0269881114565144

[72] Johnson, M. W. Classic psychedelics in addiction treatment: the case for psilocybin in tobacco smoking cessation. *Curr. Top. Behav. Neurosci.* **56**, 213–227 (2022). 35704271 10.1007/7854_2022_327

[73] Guss J., Krause R. & Sloshower, J. *The Yale Manual for Psilocybin-Assisted Therapy of Depression (using Acceptance and Commitment Therapy as a Therapeutic Frame)*. (2020).

[74] Payne, J. E., Chambers, R. & Liknaitzky, P. Combining psychedelic and mindfulness interventions: synergies to inform clinical practice. *ACS Pharmacol. Transl. Sci.* **4**, 416–423 (2021). 33860171 10.1021/acsptsci.1c00034 PMC8033772

[75] Yaden, D. B. et al. Psychedelics and psychotherapy: cognitive-behavioral approaches as default. *Front. Psychol.* **13**, 873279 (2022). 35677124 10.3389/fpsyg.2022.873279 PMC9169963

[76] Brennan, W. & Belser, A. B. Models of psychedelic-assisted psychotherapy: a contemporary assessment and an introduction to embark, a transdiagnostic, trans-drug model. *Front. Psychol.* **13**, 866018 (2022). 35719571 10.3389/fpsyg.2022.866018 PMC9201428

[77] Studerus, E., Kometer, M., Hasler, F. & Vollenweider, F. X. Acute, subacute and long-term subjective effects of psilocybin in healthy humans: a pooled analysis of experimental studies. *J. Psychopharmacol.* **25**, 1434–1452 (2011). 20855349 10.1177/0269881110382466

[78] Modlin, N. L. et al. Optimizing outcomes in psilocybin therapy: considerations in participant evaluation and preparation. *J. Affect Disord.* **326**, 18–25 (2023). 36707036 10.1016/j.jad.2023.01.077

[79] Greń, J. et al. Call for evidence-based psychedelic integration. *Exp. Clin. Psychopharmacol.* **32**, 129–135 (2024). 38010760 10.1037/pha0000684

[80] de Vos, C. M. H., Mason, N. L. & Kuypers, K. P. C. Psychedelics and neuroplasticity: a systematic review unraveling the biological underpinnings of psychedelics. *Front. Psychiatry* **12**, 724606 (2021). 34566723 10.3389/fpsyt.2021.724606 PMC8461007

[81] Wall, M. B. et al. Neuroimaging in psychedelic drug development: past, present, and future. *Mol. Psychiatry* **28**, 3573–3580 (2023). 37759038 10.1038/s41380-023-02271-0 PMC10730398

[82] Goodwin, G. M., Malievskaia, E., Fonzo, G. A. & Nemeroff, C. B. Must psilocybin always “assist psychotherapy”? *Am. J. Psychiatry* **181**, 20–25 (2024). 37434509 10.1176/appi.ajp.20221043

[83] Wagner, A. C., Mithoefer, M. C., Mithoefer, A. T. & Monson, C. M. Combining cognitive-behavioral conjoint therapy for PTSD with 3,4-methylenedioxymethamphetamine (MDMA): a case example. *J. Psychoact. Drugs* **51**, 166–173 (2019). 10.1080/02791072.2019.1589028 30890035

[84] Pahnke, W. N., Kurland, A. A., Unger, S., Savage, C. & Grof, S. The experimental use of psychedelic (LSD) psychotherapy. *JAMA* **212**, 1856–1863 (1970). 5467681

[85] Sherwood, J. N., Stolaroff, M. J. & Harman, W. W. The psychedelic experience-a new concept in psychotherapy. *J. Neuropsychiatry* **4**, 69–80 (1962). 13977209

[86] Sloshower, J. et al. Psilocybin-assisted therapy of major depressive disorder using acceptance and commitment therapy as a therapeutic frame. *J. Context. Behav. Sci.* **15**, 12–19 (2020).

[87] Ross, S. et al. Rapid and sustained symptom reduction following psilocybin treatment for anxiety and depression in patients with life-threatening cancer: a randomized controlled trial. *J. Psychopharmacol.* **30**, 1165–1180 (2016). 27909164 10.1177/0269881116675512 PMC5367551

[88] Johnson, M. W., Garcia-Romeu, A., Cosimano, M. P. & Griffiths, R. R. Pilot study of the 5-HT2AR agonist psilocybin in the treatment of tobacco addiction. *J. Psychopharmacol.* **28**, 983–992 (2014). 25213996 10.1177/0269881114548296 PMC4286320

[89] Stauffer, C. S., Anderson, B. T., Ortigo, K. M. & Woolley, J. Psilocybin-assisted group therapy and attachment: observed reduction in attachment anxiety and influences of attachment insecurity on the psilocybin experience. *ACS Pharmacol. Transl. Sci.* **4**, 526–532 (2020). 33860182 10.1021/acsptsci.0c00169 PMC8033604

[90] Carhart-Harris, R. L. et al. Psilocybin with psychological support for treatment-resistant depression: an open-label feasibility study. *Lancet Psychiatry* **3**, 619–627 (2016). 27210031 10.1016/S2215-0366(16)30065-7

[91] Doss, M. K. et al. Psilocybin therapy increases cognitive and neural flexibility in patients with major depressive disorder. *Transl. Psychiatry* **11**, 574 (2021). 34750350 10.1038/s41398-021-01706-y PMC8575795

[92] Gasser, P., Kirchner, K. & Passie, T. LSD-assisted psychotherapy for anxiety associated with a life-threatening disease: a qualitative study of acute and sustained subjective effects. *J. Psychopharmacol.* **29**, 57–68 (2015). 25389218 10.1177/0269881114555249

[93] Palhano-Fontes, F. et al. Rapid antidepressant effects of the psychedelic ayahuasca in treatment-resistant depression: a randomized placebo-controlled trial. *Psychol. Med.* **49**, 655–663 (2019). 29903051 10.1017/S0033291718001356 PMC6378413

[94] Mithoefer, M. C. et al. MDMA-assisted psychotherapy for treatment of PTSD: study design and rationale for phase 3 trials based on pooled analysis of six phase 2 randomized controlled trials. *Psychopharmacology* **236**, 2735–2745 (2019). 31065731 10.1007/s00213-019-05249-5 PMC6695343

[95] Mitchell, J. M. et al. MDMA-assisted therapy for severe PTSD: a randomized, double-blind, placebo-controlled phase 3 study. *Nat. Med.* **27**, 1025–1033 (2021). 33972795 10.1038/s41591-021-01336-3 PMC8205851

